# The evolution of the concept of synesthesia in the nineteenth century as revealed through the history of its name

**DOI:** 10.1080/0964704X.2019.1675422

**Published:** 2019-11-08

**Authors:** Jörg Jewanski, Julia Simner, Sean A. Day, Nicolas Rothen, Jamie Ward

**Affiliations:** a Institute of Musicology, University of Vienna, Vienna, Austria; b Department Musikhochschule, University of Münster, Münster, Germany; c School of Psychology, University of Sussex, Brighton, UK; d Department of Behavioral and Social Sciences, Trident Technical College, Charleston, South Carolina, USA; e Faculty of Psychology, Swiss Distance Learning University, Brig, Switzerland

**Keywords:** Synesthesia, audition colorée, color hearing, Farbenhören, nineteenth century, terminology, philology, history of medicine, history of psychology

## Abstract

Synesthesia is a rare perceptual condition causing unusual sensations, which are triggered by the stimulation of otherwise unrelated modalities (e.g., the sensation of colors triggered when listening to music). In addition to the name it takes today, the condition has had a wide variety of designations throughout its scientific history. These different names have also been accompanied by shifting boundaries in its definition, and the literature has undergone a considerable process of change in the development of a term for synesthesia, starting with “obscure feeling” in 1772, and ending with the first emergence of the true term “synesthesia” or “synæsthesiæ” in 1892. In this article, we will unpack the complex history of this nomenclature; provide key excerpts from central texts, in often hard-to-locate sources; and translate these early passages and terminologies into English.

## Introduction

The condition we know today as synesthesia (UK spelling: *synaesthesia*) is a rare involuntary trait. People with synesthesia report extraordinary “phantom” sensations, such as colors or tastes, triggered by everyday activities such as reading or listening to music. Synesthesia is a phenomenon with many different forms. Sean A. Day (2019) listed 73 different types of synesthesia, based on self-descriptions of 1,143 persons. The five most frequent forms are graphemes to colors, time units to colors, musical sounds to colors, general sounds to colors, and phonemes to colors. Despite their variety, all kinds share certain defining characteristics: They tend to be automatic, are consistent over time, present from early childhood, run in families, and are experienced by approximately 4% of the population (for an overview, see Simner and Hubbard 2013; cf. Simner 2012a; Cohen Kardosh and Terhune 2012; Eagleman 2012; Simner 2012b).

Synesthesia has received a number of different names throughout history. In this historical overview, we unscramble this history in 10 steps, considering its nomenclature from 1772 until 1892 (with an additional overview of the twentieth and twenty-first centuries), when the term synesthesia was used for the first time in a way we would recognize it today. Variants of the same lexical term (e.g., *sunaisthesis*) had been used by ancient Greek and Latin scholars in a variety of unrelated contexts (including some medical contexts) but not to refer to the phenomenon of synesthesia as we understand it today (cf. Adler and Zeuch 2002; Flakne 2005; von der Lühe 1998, 768; Schrader 1969, 46–49). We do not consider these earlier usages here. In our descriptions below, we always cite the term for synesthesia in its original language; if the original term is not in English, we add an English translation in squared brackets.^1^

The article includes historical sources from German, Latin, French, English, Italian, Swedish, Russian, and Spanish. In the period to be discussed, from the late eighteenth until the early twentieth century, no sources from South America (with the exception of Mercante 1908, 1909, 1910), Africa, Asia, or even from Australia are known today (cf. the main bibliographies, discussed in Jewanski 2013, 390–391).

## From *obscure feeling* (1772) to *pseudochromesthésie* (1864)

Although the first documented synesthete in history was the Austrian Georg Tobias Ludwig Sachs (1786–1814; publication: 1812; cf. Jewanski, Day, and Ward 2009, Jewanski, Ward, and Day 2012; Jewanski, Ward, and Day 2014), we have an earlier, albeit less than concrete, quote from the German poet and philosopher Johann Gottfried Herder (1744–1803), taken from his *Treatise on the Origin of Language*:

I am familiar with more than one example in which people, perhaps due to an impression from childhood, by nature could not but through a sudden onset immediately associate with this sound that colour, with this phenomenon that quite different, obscure feeling, which in the light of leisurely reason’s comparison has no relation with it at all—for who can compare sound and colour, phenomenon and feeling? (Herder 1772, 94–95; cited after the English translation in, 2002, 106)

Herder’s formulations “could not but” and “immediately” are criteria for synesthesia, in that these indicate the automatic evocation that characterizes synesthetic sensations. And Herder’s descriptions *schnelle Anwandelung* (sudden onset) and especially *dunkle[s] Gefühl* (obscure feeling)^2^ fit with those of the early synesthete Sachs, who would write about synesthesia 40 years later. Sachs did not give a specific name to the phenomenon in his self-description as a synesthete but wrote, in Latin, about *phenomena* (features), *obscura repraesentatio* (obscure ideas), and *ipsam repraesentationem coloratam videri* (that a colored idea appears to him; Sachs 1812, 80–81). Neither did he give an explanation of the phenomenon, but the opening sentence of the paragraphs dealing with synesthesia implies that he considered it a product of the mind, and not of the eye, as some subsequent authors did (see below):

Although I am unwilling to speak anything about the *minds* [*animo*] of our albinos, yet I should nevertheless state some observations concerning *colors*. (Sachs 1812, 80, cited after the translation in Jewanski, Day, and Ward 2009, 297)

The reviewers of Sachs’ book used the expression *farbige Erscheinung* (colored manifestation; Anonymous 1813, 236) or wrote that Sachs *gewisse Dinge als farbige Gegenstände auf eigene Weise repräsentirt* (represents special things as colored objects in his own way; Anonymous 1814, 12). A German translation of Sachs’s book from 1824 translated *phenomena* with *Erscheinungen, obscura repraesentatio* with *dunkle Vorstellung*, and *ipsam repraesentationem coloratam videri* with *daß ihm eine gefärbte Vorstellung erscheine* (Sachs 1812, transl. 1824, 99).

During the period 1815–1847, we know of no sources about synesthesia. However, in 1848 a new movement in science began that would coin several terms for synesthesia during the following decades. Its earliest terms derived from the word for color, because all known synesthetes at that time had a form of synesthesia in which various stimuli (e.g., hearing music, reading letters) triggered color sensations. The first term was provided by the French physician Charles-Auguste-Édouard Cornaz (1825–1911) in his medical dissertation about eye diseases; he named it *hyperchromatopsie* (*perception de trop de couleurs*) (hyperchromatopsia [perception of too many colors]; Cornaz 1848, 150; cf. Jewanski et al. 2012). This was because Cornaz viewed the condition as somehow opposite to the known condition of *chromatodysopsie* (chromatodysopsia: color blindness). Concerning the perception of colors, Cornaz regarded color blindness (then known as Daltonism) as some type of *an*esthesia (absence of sensation), and therefore created the analog *hyperchromatopsie* as representing *hyperesthésie du “sens des couleurs”* (hyperesthesia of the “color sense”; Cornaz 1848, 150). In 1851, he named an article with this term: *De l’hyperchromatopsie* (Cornaz 1851). This term is close to our name today, synesthesia (hyper-esthesia: hyper sensation; syn-esthesia: combined/united sensations). The term Cornaz had invented found its way into an American dictionary entry in the 15th revised edition of the often-published *Medical Lexicon. A Dictionary of Medical Science*:

HYPERCHROMATOPS’IA, Hyperchromatop’sy, from hyper, χρωμα χρωματος, “colour,” and οψις, “vision.” A defect of vision, owing to which ideas of colour are attached to objects, which convey no such coloured impressions to a healthy eye. It is the antithesis to achromatopsia. (Dunglison 1857, 480)

This dictionary entry was reprinted in all the following editions in the United States until 1874 (Dunglison 1874, 521), but still did not appear in the 1856 edition (Dunglison 1856). Therefore, nine years after the first term for synesthesia had already been coined in Europe, it was first adopted in the United States, becoming the first published source about synesthesia in that country, albeit hidden in a medical lexicon, and it was reprinted several times during the next nearly two decades.

In 1864, the French physician Chabalier gave the condition a new name, which emphasized that (for him) it was a disturbance of vision. He named it, therefore, *pseudochromesthésie* (pseudochromesthesia), because of the perception of false colors (Chabalier 1864). From 1864 onward, Cornaz’s term from 1848 fell out of use. Both Cornaz and Chabalier took pains to use their terms not only in articles or monographs but also more prominently in their titles, which made it easier for followers to catch on to their new term.

Cornaz’s eye-based explanation of synesthesia, which had led to his new term, was criticized even during the 1850s, when scientific developments of a synesthesia concept started:

In the present state of our knowledge we are not in a position to offer any satisfactory explanation of this singular anomaly of vision. That its seat is not in the eye but in the sensorium is however most probable. (White Cooper 1852, 1462)

An anonymous reviewer of Cornaz’s article specified:

[T]he author may be wrong in his assumption that we are dealing with something that is opposite to Daltonism. Instead, this strange phenomenon is likely to be based on a mapping between a sensuous perception and a certain physiological conception. (Anonymous 1852)

## *Synésthesie* (1864), or our modern term for something different

In the year of Chabalier’s article, 1864, the term *synesthésie* was used by the famous French physiologist (Edmé Félix) Alfred Vulpian (1826–1887). He inserted it in a public lecture at the end of his 20th *Lecon sur la physiologie*, dated July 21, and published these lectures two years later (1866, 465; cf. Schrader 1969, 46–49). But Vulpian’s understanding of synesthesia was different from ours today. He used it specifically for phenomena linking touch or light to coughing or sneezing (see below), which he related to the tail of the medulla oblongata (in the brainstem) in particular. It certainly had no links at that time to *hyperchromatopsie* or *pseudochromesthésie* in this context:

Mechanical irritation of the external auditory canal gives rise to a special sensation, a tickling in the throat, that makes people cough. The impression on the eyes of a bright light, sunlight for example, causes a particular tickle in the mucus membrane in the nasal cavity and indirectly provokes a fit of sneezing in certain susceptible people. … It’s via the terms *sympathy* [original: *sympathie*] or *synesthesia* [original: *synesthésie*] that we must designate the phenomena in question. Or even, with Müller, we could use the expression *associated sensations* [original: *sensations associées*]. (Vulpian 1866, 463 and 465)

Vulpian referred to the German physiologist Johannes Müller (1801–1855), who had named these same phenomena *Mitempfindungen* (cosensations; Müller 1837, 708; but not yet in his earlier book *Ueber die phantastischen Gesichtsempfindungen* [*On Phantastic Appearances of the Visual Sense*], 1826). These Mitempfindungen had been observed at least 100 years ago. For the English clergyman Stephen Hales, famous for his measurements of blood pressure, they were examples of the “sympathy of the nerves” (1733, 60). Vulpian created the term *synesthésie*, probably analogous to the terms *anesthésie, thermesthésie*, and *hyperesthésie* (Schrader 1969, 47), which he later used in an article “Moelle (Physiologie)” (“Spinal Cord [Physiology]), which had a separate chapter *Synesthésies* (Vulpian 1874, 519–527).

Therefore, in 1864, the term synésthesie was used with our modern spelling, but with a different meaning. What makes it more complicated is that this term in 1864 had also been used as a synonym for *Mitempfindungen*. And, in turn, during the 1880s, *Mitempfindungen* was used as a synonym for synesthesia in our modern sense (Hilbert 1884), as we will see later.

## From subjective *Farben-Empfindungen* (1873) to *Secundärempfindungen* (1881)

Outside of these discussions, the American poet Hannah Reba Hudson (1873; cf. Jewanski et al. 2011, 301–302) named her own number-to-form synesthesia *idiosyncrasy* rather than finding a name deriving from the sensations themselves. Her article was not published in a medical journal, but in a magazine for literature, art, and politics, and was rarely noticed by others. It was, however, rediscovered for synesthesia research by Francis Galton (1880a), although he did not adopt her term.

Also in 1873, the Austrian synesthete Fidelis Alois Nussbaumer (1848–1919) described our phenomenon as “subjective ‘*Farben’empfindungen*” (subjective “color” sensations; Nussbaumer 1873a; cf. Jewanski et al. 2013), also with the different spelling “subjective *Farben-Empfindungen*” (Nussbaumer 1873b). They derived the term from their own (musical and general) sound to vision-synesthesia. Two months later, in a related article, Nussbaumer suggested a new name: *Phonopsie* (phonopsia) for *Töne-Sehen* (seeing sounds; Nussbaumer 1873b, 60).

At this point in history, the earlier cases of synesthesia and the different terms they had once had were all but forgotten. This is largely because Nussbaumer regarded himself as being the first synesthete in history and the first to give it a name. His point of view was adopted by his subsequent followers from various countries. (The earlier cases of synesthesia and terms used for them were only rediscovered in 1890, by Suarez de Mendoza.) Nussbaumer’s new term *Phonopsie* was published in an obscure journal, *Mittheilungen des Aerztlichen Vereines in Wien* (*Communication of the Association of Physicians in Vienna*), nothing more than a newsletter for a local association; so it was rarely noticed by others. The antiquated idea of an eye-based explanation of synesthesia was still in use: The ophthalmologist Jakob Hock struck a tuning fork and in vain tried to observe changes in Nussbaumer’s retina or optic nerve (Hock 1873).

Up to 1873, only a few cases of synesthesia were known (a list appeared in Jewanski 2013, 373, which can be enlarged with at least one more recently rediscovered early case: a man who saw synesthetic colors to the voices of 24 famous singers of his time: Lumley 1864, 98–99). From 1876 on, the leading thinkers of their time joined the discussion and developed theories about a synesthesia concept, which is also reflected in the names for the phenomenon, and the reported cases jumped from only a few to more than 100. The transition from single cases toward large-scale surveys does represent an important historical marker: For example, Suarez de Mendoza published a list of 134 inducer-to-vision synesthetes, compiled from 36 sources and complemented with eight new cases (Suarez de Mendoza 1890, Appendix). We will come back to him later.

In 1876, the German psychologist Gustav Theodor Fechner (1801–1887) analyzed color associations mainly for vowels, and collected 442 cases of color associations, but he did not differentiate between synesthetes and nonsynesthetes, and therefore did not use a specific term for synesthesia (Fechner 1876; cf. Jewanski et al. 2019, 2–4).

The British philosopher George Henry Lewes (1817–1878), while discussing Nussbaumer’s case, replaced Nussbaumer’s adjective “subjective” with the word “double,” thereby creating the term “double sensation,” to which he devoted an entire book chapter (Lewes 1879, 280–287). His term was, by its very meaning, rather close to our term today: double sensation, meaning combined or united sensations (synesthesia). However, when synesthesia was subsequently described by Sir Francis Galton (1822–1911; e.g., Galton 1880b; cf. Jewanski et al. 2019, 4–7), Lewes’s monograph was not mentioned at all. Although Galton was to some extent interested in synesthesia for color, he was principally interested in “number forms” (known today as *sequence-space synesthesia*, in which synesthetes associate numbers with specific locations in space), or perhaps in what we know today as *sequence-personality synesthesia* (in which numbers are associated with personifications; e.g., seven is a shy male). Galton saw these phenomena as part of unusual mental imagery, in the same category as visual hallucinations, visual memory of scenes, or hypnagogic imagery. For this reason, perhaps, Galton did not provide a special term for the phenomenon (cf. Jewanski et al. 2019, 4–7), but instead named people possessing it as “seers,” whereas other people were “non-seers” (Galton 1880b, 495).

The Swiss academics Eugen Bleuler (1857–1939) and Karl Bernhard Lehmann (1858–1940), who later became famous scientists—Bleuler as a psychiatrist, Lehmann as a hygienist—were medical students when they discovered six different kinds of synesthesia (Bleuler and Lehmann 1881, 3–4):

1. sound photisms: light, color, and form sensations which are elicited through hearing;
2. light phonisms: sound sensations which are elicited through seeing;
3. gustation photisms: color sensations for gustation perceptions;
4. olfactory photisms: color sensations for olfactory perceptions;
5. color and shape sensations for pain, heat, and tactile sensations; and
6. color sensations for shapes.

The most frequent of these they believed was No. 1. Bleuler and Lehmann rejected Nussbaumer’s term *Phonopsie*, because it covered only parts of the topic, and the same was true for *Farben-Empfindungen*, because “light” was more than simply “color” (Bleuler and Lehmann 1881, 4, note). Bleuler and Lehmann also rejected the term *Mitempfindungen* simply to avoid misunderstandings (Bleuler and Lehmann 1881, 3, note). Here, they were probably referring to the term as used by Müller (see above).

Instead of using these earlier terms, Bleuler and Lehmann (1881, 3, note) named the phenomenon *Secundärempfindungen oder Secundärvorstellungen* (secondary sensations or secondary imaginations. This appears to be because they were not sure whether the phenomenon dealt with experiences that were perceived or imagined, although they were more in favor of the former. Bleuler and Lehmann’s book (1881) was entitled *Lichtempfindungen* (*Light Sensations*), as the most frequent form of synesthesia, and in its subtitle they integrated “*verwandte Erscheinungen*” (“related phenomena): *Zwangsmässige Lichtempfindungen durch Schall und verwandte Erscheinungen auf dem Gebiete der andern Sinnesempfindungen* (*Compulsory Light Sensations Through Sound and Related Phenomena in the Domain of Other Sensations*). Although Bleuler and Lehmann had named the phenomenon *Secundärempfindungen* respectively *Secundärvorstellungen* and coined a new term, they did not use this term in the title of their book.

Bleuler and Lehmann developed several features of synesthesia (cf. Jewanski et al. 2019, 7–10): They described for the first time the plurality of synesthesia; emphasized a continuum between people with and without synesthesia; regarded synesthesia as a kind of atavism; showed that synesthesia was not linked to mental illness, as it was regarded in the context of Nussbaumer; and considered the phenomenon as being “existent in the predisposition of everyone” (pp. 50–51); among 596 people, they discovered a frequency of 12.8% synesthetes (which is much higher than today’s accepted 4%; Simner et al. 2006). Their term *Sekundärempfindungen* respectively *Secundärvorstellungen* derived from Nussbaumer’s term subjective *“Farben”empfindunge*n. Due to the features of synesthesia Bleuler and Lehmann had developed, they removed the word “color” and replaced “subjective” with “secondary,” which is a more neutral term and put the phenomenon far away from an individual, subjective mental illness.

## Farbenhören – Colo(u)r hearing – audition colorée (1881/82), or the name of one type of synesthesia became the name of the whole phenomenon

Bleuler and Lehmann’s book was reviewed in July 1881 in the Austrian regional newspaper *Neue Freie Presse* (*New Free Press*), published in Vienna, under the headline “Das Farbenhören” (“Color Hearing”). This term, still used sporadically today, was invented by the reviewer (“a new built word”; Anonymous 1881a, left col.), since the term *Farbenhören* had not been used in Bleuler and Lehmann’s book itself (this is because Bleuler and Lehmann had taken pains to expand the use of “color” to “light”). The term *Farbenhören* in fact means “in Farben hören” (hearing in colors; Anonymous 1881a, left col.) and was probably chosen because Bleuler and Lehmann had described sound photisms (light, color, and form sensations that are elicited through hearing) as the most frequent form. Nonetheless, it is important to note that this new term not only meant a stimulus-to-color-synesthesia but was used for the whole phenomenon we today name synesthesia.

A month after this review appeared, it was reprinted again in the German medical journal *Medizinische Neuigkeiten für praktische Ärzte* (*Medical News for Practitioners*) and therefore reached physicians (Anonymous 1881b). (By chance, this journal was published in Erlangen, the same city where Sachs had received his doctoral degree.) In October 1881, in the American medical journal *The Cincinnati Lancet and Clinic, a Weekly Journal of Medicine & Surgery*, the German review was translated and reprinted yet again, this time inside a section called “Ophthalmology and Otology.” Here, the English term “color hearing,” a translation of the German *Farbenhören*, appeared for the first time (Anonymous 1881c).

The journey of this review from Vienna to Erlangen to Cincinnati, Ohio, finally ended in London, where the American version of the article again was reprinted in December 1881 in *The London Medical Journal*. Here, the title was transferred to “Colour-Hearing” (Anonymous 1881d). This reprint in *The London Medical Journal* was known to the Frenchman Louis-Marie-Alexis Pédrono (1859–1942), an assistant at an ophtalmological clinique who, in 1882, published twice an article with the title *De l’audition colorée* (1882a, 1882b), the first French translation of “Colo(u)r(-)Hearing,” With this introduction by Pédrono, France was to become the most important nation for research on synesthesia during the following decade (Capanni 2019, in preparation).

We can see that the years 1881 to 1882 saw a somewhat complex development in the naming of synesthesia: One single article (Anonymous 1881a) led to a new term in German, American English, British English, and French. Important for us here is that all of these terms meant, during the 1880s, what we today name synesthesia, whereas today, conversely, these historical names are used only for one *variant* of synesthesia in particular: sound-to-color synesthesia.

Although Pédrono was aware of Bleuler and Lehmann’s monograph, he reduced the phenomenon again to a music-to-color-synesthesia, translated the term *Farbenhören* to “*audition colorée*” and offered an explanation, which was valid only for this form of synesthesia:

[W]e allow the theory of irradiation or of associated sensations, we can say that the centers of color and sound are necessarily situated close to each other. … Whether one supports the idea of an abnormal pathway of nerve fibers coming from the ear, or whether one supports the theory of irradiation, the final phenomenon is excitation associated to the central acoustic and color cells. (Pédrono 1882a, 308 and 310; 1882b, 235 and 237)

This theory of an anatomical closeness of brain regions had been developed in Italy in 1873 by the physiologist Filippo Lussana (1820–1897; Lussana 1873, 122; cf. Jewanski et al. 2011, 299–301).

The Italian translation *udizione colorata* of Pédrono’s term was used by the physician Vittorio Grazzi (Grazzi 1883); in 1890, a Swedish translation was published as *färghörsel* (Wahlstedt 1890). Two years later, the German social critic Max Nordau (1849–1923) published the monograph *Entartung* (*Degeneration*; 1892), in which he regarded synesthesia as some kind of degenerative trait; he felt the same way about other types of cross-modal correspondences shared by the population at large (for a review of literature on cross-modal correspondences, see Spence 2011).

Nordau’s book was translated into several languages (e.g., 1894 into Russian and 1902 into Spanish), and therefore introduced the term *audition colorée* for the first time to Russian as *воспринимание слухом цветов* (Nordau 1894, 144), as well as to Spanish as *audición coloreada* (Nordau 1902, 218).

The term color hearing is ambiguous and can mean “hearing in colors” (i.e., sound triggers colors) or “hearing the sound of colors” (i.e., colors trigger sound; i.e., an incorrect interpretation, at least with reference to the examples given in these earlier works). As a synonym for color hearing, sometimes colored hearing (Billings 1888) was used, and even a mixing of languages as color-audition (Color-audition 1888) or colored audition (Binet 1892a; 1893); that is, a blend of English and French.

In 1889, a conference took place in Paris that had particular significance for synesthesia researchers. At the *Congrès international de psychologie physiologique*, there was a separate session on “audition colorée,” with a panel that included the psychologists Théodore Flournoy, from Switzerland, and Eduard [Édouard] Gruber, from Romania. This forum promoted the use of the term *audition colorée* to denote all kinds of synesthesia (Société de psychologie physiologique 1890; cf. Jewanski et al. 2015; 2019, 11–15):

The congress expresses the wish that it proceeds to an enquiry on the phenomena named *audition colorée* [colored hearing], taking this term in the most general of constant link between the sensations of diverse senses. (Société de psychologie physiologique 1890, 157)

With this goal in mind, it confirmed the terminological development of the term for synesthesia during the 1880s. As a direct result of this conference, three books about synesthesia were published within three years in France (Flournoy 1893; Millet 1892; Suarez de Mendoza 1890); all of them had the term *audition colorée* in the title, and meant what we today name broadly as synesthesia. We will later return to each of these authors.

## Germany: Variants of *Empfindungen* (1880s)

During the 1880s, where synesthesia was discussed in German circles, authors used various instances of Bleuler and Lehmann’s term *Secundärempfindungen* and always used the word *Empfindungen* within their description. Although Bleuler and Lehmann had avoided the term *Mitempfindung* (cosensation; 1881, 3, note), physician Richard Hilbert (1884, 3; followed by Quincke 1890) used it. In his article, Hilbert alternated between *Doppelempfindungen* and *Mitempfindungen* and used both of them synonymously. Hermann Steinbrügge (1887) titled his inaugural speech as a professor of medicine using the words “secundäre Sinnesempfindungen” (secondary sensations of senses). Two German medical dissertations about synesthesia were published in the next two years: Ludwig Deichmann (1889) named it *secundäre Empfindungen* (secondary sensations) and Albert Ellinger (1889) called it *Doppelempfindung (Secundärempfindung)* (double sensation [secondary sensation]).

In German texts, one exception to using derivatives of Beuler and Lehmann’s terms came from the physician Arthur Sperling (1860–?), who was also present at the 1889 Paris conference. Sperling named it in French *chromatopsie* (chromatopsia; cited from Société de psychologie physiologique 1890, 95). This term was nothing new, and at that time it was used for any anomaly of visual perception and also as a synonym for *chromopsia*. Sperling’s integration of synesthesia into a normal medical term passed unnoticed by others, because it was only one short remark within a discussion.

## *Synesthésie* versus audition *colorée* (1888)

One of the German variants of *Empfindungen* used to describe synesthesia had been *Mitempfindungen* (Hilbert 1884). A historical study about the use of these *Mitempfindungen* (but in the tradition of Müller’s use of the term, who had used them for bodily reflexes; see above) was carried out in Russia by the physician Nicolaj Kovalevskij (1884). Kovalevskij was the first to use the term *Mitempfindung* in Russia (Vanečkina, Galeev, and Galjavina 2005). His article found its way to the West two years later via a shortened translation into German (Nawrocki 1886).

In 1888, the French physician Henry de Fromentel regarded *synésthesie* as an “(unspecified) double sensation (original: double sensation) that the subject experiences in two distinct points of the body, more or less separated from each other, under the influence of excitation carried by one of these points” (de Fromentel 1888, 9). Fromentel took Chabalier’s term *pseudochromesthésie* (1864) as an example, and regarded the phenomenon as a *subdivision des synesthésies* (subdivision of synesthesiae; de Fromentel 1888, 172).

Two years after that, the French physiologist Henri-[Henry-]Étienne Beaunis (1830–1921) used the term *sensations associées* (associated sensations), which he divided into two groups. For him, phenomena described by Kovalevskij and Fromentel belonged to a first group and were named *synalgies* and *synesthésies*; phenomena described by Bleuler and Lehmann belonged to a second group, which he named *audition colorée*:

In a first group, the secondary sensation has the same quality as the primary sensation. So, a tactile excitation would evoke a secondary tactile sensation at a point on the organism that was not excited. For example, the touch of an external auditory conduit near the tympanic membrane determines a tickling sensation in the larynx. We can include in this category associated sensations [original: sensations associées] called *synalgies* and *synesthesias* [original: *synalgies* and *synesthésies*] of which Fromentel and Kovalevskij have furnished the typography. … In a second group, the secondary sensation is qualitatively different to the primary sensation. It’s within this category that the very curious fact of *colored hearing* [original: *audition colorée*] belongs. (Beaunis 1888, 795 and 796)

**Figure 1.**
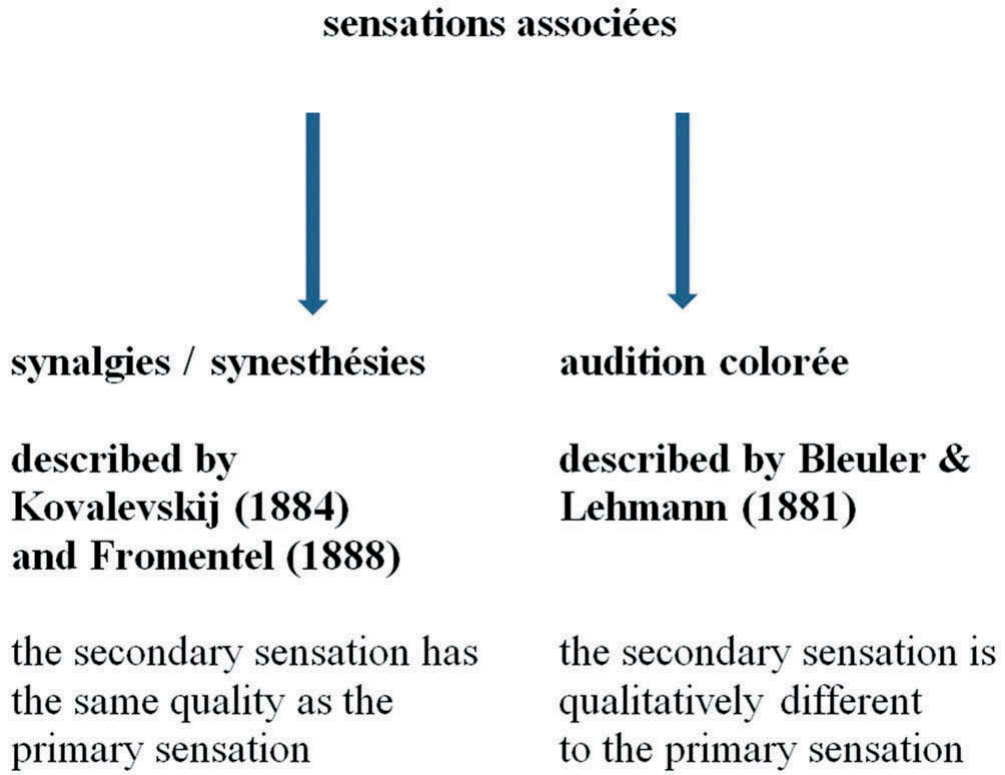
Sensations associées (Beaunis 1888). This figure shows the interpretation of terms used by Beaunis, relating different types of paired sensations.

We can represent his views within a chart (Figure 1) to simplify the complex issue.

Our modern use of the term synesthesia appears on the right side, but is named—in the French tradition of the 1880s—as *audition colorée*. Our modern term synesthesia appears on the left side, but means bodily reflexes, which also were named cosensations respectively *synalgies*. Cosensation at that time in Germany was also a synonym for synesthesia as we use it today. The higher-ordered term used by Beaunis was *sensations associées*, which can be translated as associated sensations, or as cosensations. The latter translation refers to *synalgies/synesthésies* as used by Beaunis, but etymologically a cosensation is a “syn-esthesia”.

In Germany, in 1890, the physician Heinrich Quincke (1842–1922) subdivided *Mitempfindungen* (in the meaning of Müller) into six groups: No. 1 was *Miterregung sensibler Bahnen (Mitempfindung)* (co-stimulation of sensible pathways], No. 2–6 dealt with reflexes; No. 5 was *Nervöse Vorgänge, welche mit Vorstellungen in Beziehung stehen* (nervous processes, which are related to imaginations). Parts of synesthesia belong to Nos. 2 and 5; but, for Quincke, it was unclear whether the phenomenon was imagination or a perception. This was a question that had also been left unanswered by Bleuler and Lehmann (1881).

The relation between synesthesia (in our modern meaning) and *synalgies* (bodily reflexes) was not clarified until somewhat recently. Burrack, Knoch, and Brugger (2006) were interested in the latter phenomenon, in which tactile stimulation of one part of the body leads to a sensation in another zone (in this context, *Mitempfindungen* is translated as “referred itch”; Evans 1976). Burrack et al. found the prevalence of *Mitempfindung* (they used the German term) in synesthetes at 40%, compared to only 10% in a control group. Burrack et al. noted several similarities between both phenomena: Both originate in early childhood; both often run in families; the relation between the trigger and the reference zone is stable over time within an individual, but very variable across individuals; and both are unidirectional (e.g., for any synesthete, sounds tend to trigger colors, but colors do not trigger sounds).

## *Synesthésie* (1892)–*Synæsthesiæ* (1892)

In 1890, one year after the Paris conference noted above, the Puerto Rican ophthalmologist and otologist named Ferdinand Suarez de Mendoza (1852–1918), who worked mainly in Paris, described synesthesia using the term *fausse sensations secondaire* (false secondary sensations). Suarez de Mendoza was likely influenced by Chabalier’s term *pseudochromesthésie* (1864), but expanded it to five different kinds, each based on one sense (Suarez de Mendoza 1890, 8):

- *La pseudophotesthésie* (false optic sensations)
- *La pseudo-acouesthésie* (false aural sensations)
- *La pseudosphrésesthésie* (false olfactory sensations)
- *La pseudogousesthésie* (false gustatory sensations)
- *La pseudo-apsiesthésie* (false tactile sensations)

In the United States, research on synesthesia started in 1892. Here the psychologist William O. Krohn (1868–1927), a founding member of the American Psychological Association, again adopted Chabalier’s term from 1864, because he regarded the phenomenon, which he narrowed down to a stimulus-color synesthesia, as a “pseudo-color sensation” (Krohn 1892, 21). He spelled the term in English as pseudo-chromesthesia and gave the following explanation which integrated several ideas of previous synesthesia concepts:

Some few [cases] may depend somewhat upon the association of *ideas* dating from youth, developed in a manner conscious or unconscious. … In the greater percent of cases the pseudo chromesthesic phenomena arise from some sort of cerebral work which is the outcome of the close relation of the cortical centers, which are connected by numerous associational fibers, notably the visual and auditory centers. Whether this is done by anastomosis of fibres or irradiation, or by direct stimulus of the fibres of associations, it is evident that in some cases at least it takes place within the centers themselves. (Krohn 1892, 38)

The psychologist Mary Whiton Calkins (1863–1930), the first woman to become president of the American Psychological Association (in 1905), titled her first article on this issue using the same term “pseudo-chromesthesia” (1893a) and published this article within the same journal as Krohn.

Although scientists in the United States in 1893 were still adopting the old French term *pseudochromesthésie*, one year earlier in France, Jules Antoine Millet (1865–1892) had written his medical doctoral thesis on synesthesia, and differentiated *synesthésie* (for all kinds of combined senses) from *audition colorée*:

The term “synesthesia” [original: synesthésie] carries its meaning within itself; it is equivalent to the expression “associated sensations” [original: sensations associées]; the term “color hearing” [original: audition colorée] indicates neatly that a color sensation attaches itself to the perception of sounds. (Millet 1892, 13)

This terminological use was notably different from what the Paris committee 1889 had requested, but this is how we today use these terms. So, Millet used the same term *synesthésie* as Vulpian (1866, 1874), but changed its meaning to a definition we still use today:

The term synesthesia isn’t very recent: it has been employed for the first time, we believe, by Vulpian in 1874: Vulpian substituted it for the term “reflexive sensations” to designate the associated sensations which have their seat in the medulla. (Millet 1892, 14)

The reason for using this term was quite simple:

We do not believe in having to give currency to the more or less barbaric terms proposed by M. Suarez de Mendoza, no more to *pseudophotesthésia* than to *pseudosphrésesthésia* and to *pseudo-apsiesthésia*; despite the etymological significance of these words, we don’t want to inflict on our readers the torture of having to often spell them. We’ll use simpler words, especially to translate complicated things. (Millet 1892, 14)

Previously, it had been accepted that Millet was the first to introduce the term *synesthésie* to the describe what we today name synesthesia (Segalen 1902, 57; von Siebold 1919, 5; Wellek 1927, 16, note 27; Schrader 1969, 46; Dann 1998, 21; Gruß 2017, 59; Jewanski 2013, 378; Jewanski et al. 2018). Recently, however, we found out that, in the same year 1892, the term had also been used in its modern sense in the United Kingdom by Frederic W. H. Myers (1843–1901). Myers was a philologist and founder of the Society for Psychical Research, which dealt with thought-transference, hypnotism, somnambulism, and generally “*psychologie transcendentale*” (Sidgwick and Myers 1892, 603)—all serious fields of research at that time. Myers had been present at the Paris congress in 1889 and had been aware of the session on *audition colorée* (Myers 1890). He also attended the second psychological congress, 1892, in London (Sidgwick and Myers 1892). Myers was aware of current information from 1892: Gruber’s lecture at the London congress from August, published in the same year (Gruber 1892); Krohn’s article from October (Krohn 1892); Flournoy’s article from October (Flournoy 1892); and Paul Sollier’s article about *Gustation colorée* (colored taste) from December (Sollier 1892). In the eighth volume of the *Proceedings of the Society for Psychical Research*, which appeared in 1892, Myers published chapters III–V of his article “Subliminal Consciousness,” in which he used the term *synæsthesiæ*.

The *List of Members and Associates* (Proceedings of the Society for Psychical Research 1892, 615) is dated “December, 1892”; Myers had read parts of his Chapter V during the 54th General Meeting on October 28 and during the 55th General Meeting on December 2 (Proceedings of the Society for Psychical Research 1892, 413). The paragraphs of his article that deal with newest information from October to December 1892 are only footnotes and probably added later to the main text. We can conclude that the final version of his article was finished in December 1892. It might be that the *Proceedings* were already published in 1892, or perhaps in the beginnings of 1893 with the date 1892.

Millet’s monograph also was published in 1892. We have to trace the exact date to find out if Millet or Myers was the first to use the new term. Millet’s monograph was published twice: one print in Montpellier for the medical thesis and the other one (a commercial one) for the publisher in Paris. It was not rare that medical dissertations were published by Parisian publishers. Both prints are identical in pagination. The edition by Octave Doin in Paris was published in the last week of April 1892 (Dernières Nouvelles, 1892). Moreover, in a letter dated October 10, 1892, Binet wrote that Millet’s thesis is now published (Binet 1892b). Thus, Millet was definitely the first to use the term synesthesia in our modern meaning.

In none of the articles reviewed by Myers does the term synesthesia appear, and yet Myers does not “make a song and dance” about introducing a new term. In contrast, Millet knew that he had coined a new term and had written this explicitly; but Myers used the term naturally, as if it was well known. Thus, there might be an earlier English source for synesthesia in our modern understanding, but it is not yet discovered.

A number-form [i.e., what we know today as *sequence-space synesthesia*] is an association of an image with an idea,—presumably as entirely a result of post-natal experience as is my association of my friend’s face with his name. And so also, indeed *audition colorée*,—the perception of a definite “imaginary” or “subjective” colour in association with each definite actual sound,—may in some slighter cases be due to post-natal (mainly infantile) experience working upon an innate predisposition. But when the synæsthesiæ of which sound-seeing is only the most conspicuous example are found in fuller development;—when gradated, peremptory, inexplicable associations connect sensations of light and colour with sensations of temperature, smell, taste, muscular resistance, &c., &c.;—for M. Gruber finds that these links exist in yet unexplored variety;—then it becomes probable that we are dealing, not with the casual associations of childish experience, but with some reflection or irradiation of specialised sensations which must depend on the connate structure of the brain itself. (Myers 1892, 457)

It is very probably from this same passage that the psychologist and hallucination researcher Edmund Parish (1861–1916) adopted the term *synæsthesiæ* when he translated it into German as *Synaesthesien* (Parish 1894, 156). However, neither Myers nor Parish were received by their contemporaries as the first scientists (next to Millet 1892) to coin the term synesthesia in English or in German. And neither Myers’s nor Parish’s writings have ever been mentioned in any bibliography on synesthesia until today.

## *Synesthésie* (F) / *syn(a)esthesia* (US/UK) / *sinestesia* (I/E) / *Synästhesie* (D) / *Синестезия* (RUS) (mostly 1892–1895)

The route by which the term synesthesia spread through different languages did not begin with Myers, but instead it went from (the unknown) Millet to (the well-known) Swiss psychologist Théodore Flournoy (1854–1920), who was well aware of Millet’s monograph. For Flournoy, the term *audition colorée* was problematic, as it was too narrow for his wish to include other variants of synesthesia whose experiences were not color. This was an argument Millet also had used. Flournoy considered using respectively the terms *synesthésie visuelle* (visual synesthesia), *synesthésies auditive* (auditory synesthesia), *synesthésies olfactive* (olfactory synesthesia), and also *syncinésies* (synkinesia) to denote variants involving movement (Flournoy 1893, 6). Although strongly praising the term *synesthésie*, Flournoy ultimately (and perhaps unexpectedly) settled on the term *synopsie* (synopsia; Flournoy 1893, 6), but he allowed himself to also use the terms *audition colorée* and *synesthésie* as short-cuts, and often seemingly interchangeably with *synopsie*.

Flournoy’s wealth and thoroughness of ideas as well as his modernity make his monograph the standard one from the nineteenth century. He linked several kinds of synesthesia under one banner and one term, from colored alphabets to personifications, as we do today (cf. Jewanski et al. 2019, 15–17).

Whereas Millet’s monograph remained nearly unknown, Flournoy’s monograph was reviewed several times in several countries, mostly with detailed summarization, and it was highly praised. With these reviews, published in important journals by similarly important psychologists and researchers, the new term *synesthésie* was translated into English as *synæsthesia* (Mary Whiton Calkins 1893b, in *The American Journal of Psychology*; Jean Philippe 1894, in *The Psychological Review*; and Howard C. Warren 1896, in *The American Naturalist*) and into German as *Synästhesie* (Jonas Cohn 1895, in *Zeitschrift für Psychologie und Physiologie der Sinnesorgane*), whereas others adopted the French term *synesthésie* even outside of France (Salomon Moos 1894, in *Zeitschrift für Ohrenheilkunde*) or translated Flournoy’s term *synopsia* for visual synesthesia into English as synopsy (William James 1894, in *The Philosophical Review*).

Most of the reviewers recognized the importance of Flournoy’s work, including, for example, Howard C. Warren (1867–1934), who later became president of the American Psychological Association.

Until quite recently synæsthesia was regarded by psychologists generally as a purely artificial and fanciful association, or at least a sign of degeneracy; it has lately received considerable attention, however, and the weight of evidence goes to show that it is both natural and normal—it may even be said, a phenomenon of common occurence. (Warren 1896, 689)

Within articles themselves, or even as part of an article’s title, *synesthésie* was used in different languages starting from 1893—always relating to Millet-Flournoy, but not to Myers. Below, we discuss writings in English, Italian, German, Russian, and Spanish.

### English

Two years after her review on Flournoy’s monograph, Calkins, who became an important researcher of synesthesia, was the first to name an article “Synæsthesia” (Calkins 1895). This English term was used as a synonym for associated sensations and secondary sensations one year earlier, but not as the title of an article (Colman 1894, 795, left col.: “synæsthesiæ”; p. 851, right col.: “synæsthesia”).

### Italian

Italy has a long tradition of research on synesthesia, which goes back to the 1860s (Jewanski et al. 2011; Lorusso et al. 2012; Lorusso and Porro 2010; Riccò 1999, 2011; Tornitore 1986, 2012, 2014). The art historian Mario Pilo was aware of Flournoy’s monograph as well as other writings from the 1880s and early 1890s. He named an article “Contributo allo studio dei fenomeni sinestesici” (“Contribution for the Study of Synesthetic Phenomena”) and, inside this, he used the term *sinestesia* (1894, 140). In the same year, the psychiatrist Girolamo Mirto, who also was aware of Flournoy, titled an article titled, “Contributo di fenomeni di sinestesia visuale (udizione colorata)” (“Contribution to the Phenomena of Visual Synesthesia [Colored Hearing]; Mirto 1894). Both articles were reprinted and led to a wider dissemination of the term (Mirto 1895; Pilo 1922).

### German

The German translation *Synästhesie* (*Synaesthesie*) was first used in 1894 by Parish, who gave a definition of synesthesia which corresponds to ours today.

Synæsthesia, that is to say [a] constant involuntary association of a certain image or (subjective) sensory impression with an actual sensation belonging to another sense, is observed in a variety of forms. (Parish 1894, 156, cited after the English translation in, Parish 1894, ed. 1897, 223)

By 1895, at least four German reviews (two on Colman 1894: Barth 1895; Ziehen 1895; one on Flournoy 1893: Cohn 1895; one on Mirto 1894: Hager 1895) translated, respectively, the French, English, or Italian term into *Synästhesie*, respectively, *Synaesthesie*. The first article with the term *Synästhesie* in its title was published nearly a decade later by Helene Friederike Stelzner, one of the first female physicians with a medical doctor degree in Germany, based on a self-description (Stelzner 1903).

### Russian

The first to use the term *Синестезия* [synesthesia] in an article was the psychologist Pavel Petrovič Sokolov, in 1897; the first to use it in the title of an article (*Синестезии* [*Synesthesiae*]) was Ivan Dmitrievič Ermakov, one of the first Russian psychoanalysts, in 1914 (Sokolov 1897, 253; Ermakov 1914; Kuznetsova 2005, 20–21; Sidoroff-Dorso 2012, 235–39; 2014, 291–95).

### Spanish

By 1908, the term synesthesia appeared as *sinestesia* in an article by the educational psychologist Victor Mercante, and was repeated by him in the following years (Mercante 1908, 422; 1909, 470; 1910, 17), as well as in 1931 in a title by the physician Juan Ramón Beltrán (Beltrán 1931). All of the journal articles were published in Buenos Aires, Argentina; Beltrán’s monograph, in Madrid, Spain.

## The acceptance of the term synesthesia since 1895

By 1895, the term synesthesia had appeared in several languages, ending a mode of thought dating back half a century which had reduced synesthesia to a pure color-based experience. Now a wide diversity of phenomena was named under the same banner of synesthesia. Still unclear were synesthesia concepts, besides the fact that the pathological and degeneration views were not pursued further. The exact kind of connections of brain areas could not be fixed, nor was the relationship to cross-modal correspondences, associations, or mental imagery clear.

Since 1895, the term synesthesia in various spellings across different languages was introduced in French, English, German, and Italian; however, it did not become accepted immediately. In the first edition of Theodor Ziehen’s *Psychiatrie. Für Ärzte und Studirende* (*Psychiatry: For Physicians and Students*), synesthesia was named *Secundäre Sinnesempfindungen* (secondary sensations of senses; Ziehen 1894, 18–20), a term used by Steinbrügge in 1887. In the second revised edition, in 1902, the chapter is renamed “Synästhesien oder Sekundärempfindungen” (Ziehen 1902, 17–21); retained also in the third revised edition (Ziehen, 1904, 17–20) and the fourth revised edition (Ziehen 1911, 17–20).

In the first volume of Charles Richet’s *Dictionnaire de physiologie*, the physiologist Jean-Pierre Nuel, who in 1876 had written a chapter about the Nussbaumer case (Nuel 1876), wrote a long entry about *audition colorée* but without mentioning the new term *synesthésie* (Nuel 1895). His newest references were Suarez de Mendoza (1890) and Gruber (1892). (The ambitious *Dictionnaire* ended with Volume 10 in 1928 and the letter *Mo*; therefore, an entry on *synesthésie* could not be included.)

The German geographer Richard Hennig, who also dedicated himself to the field of psychology, published in 1896 an article called “Entstehung und Bedeutung der Synopsien” (“Formation and Meaning of Synopsiae). In its first sentence, Hennig declared *Synästhesie* and *Mitempfindungen* as synonyms, and *Synopsie* (based on Flournoy) as the visual sense subgroup (Hennig 1896, 183). His article was reviewed by Calkins, whom we met earlier as the first mediator of Flournoy’s monograph outside of France. Aside from the fact that Calkins did not see anything new in Hennig’s article, she did not mention the term synesthesia in her review (Calkins 1896).

In the fourth edition of George M. Gould’s *An Illustrated Dictionary of Medicine, Biology and Allied Sciences*, the entry synesthesia appears (Gould 1894, ed. 1898, 1448), but referring to a cosensation in Müller’s sense. Synesthesia in the modern sense appears under different entries: *audition colorée* (p. 149), *chromesthesia* (p. 295), color hearing (p. 311), *phonopsia* (p. 1070), *Pseudo-chromestesia* (p. 1200), and some more. In these entries, the text is identical with the first edition from 1894, although the subtitle of the dictionary reads, *Based Upon Recent Scientific Literature*.

During the first years of the twentiety century, the term synesthesia became more and more established. In 1900, in the important *American Journal of Psychology*, the educational psychologist Guy Montrose Whipple named an article that dealt with colored hearing as well with pain-to-sound synesthesia and temperature-to-sound synesthesia; his title was, “Two Cases of Synæsthesia” (Whipple 1900). Two years later, under the of entry “synaesthesia” in a *Dictionary of Philosophy and Psychology*, the term also takes on our modern meaning (Titchener, Jastrow, and Baldwin 1902). In 1904, the Japanese physician Tatsusaburo Sari adopted the term and expanded it with a suffix: *Ein Fall von akustisch-optischer Synästhesie (Farbenhören)* (*A Case of Acoustic-Visual Synesthesia [Color Hearing]*); Sari 1904). One year later, the physician Henry Lee Smith used the term synesthesia again with our modern meaning (Smith 1905), as did the psychologists Arthur H. Pierce (1907) and Carl E. Seashore (1908, 115–116). In contrast, some authors still used older terms such as *sekundäre Empfindungen* (Wallaschek 1905, 149–192), which was Deichmann’s term (1889), or *Doppelempfindungen* (Utitz 1908, 73–74), which was Ellinger’s term (1889).

Articles that dealt only with color hearing (these being articles in the majority) still continued to often use terms such as *audition colorée* (even outside of France) (Abraham 1901/02, 36–39; Lach 1903), *chromæsthesia* (a new term, which could not be pushed) (Dresslar 1903), *Farbenhören* (Chalupecký 1904), or *farbiges Hören (auditio colorata)* (Lomer 1905).

## ‘*Synesthésie*’: Its artistic value and the stretching of its meaning (since 1902)

In the same vein described in our last section up to 1900, a French article in 1902 entitled *Les synesthésies et l’école symboliste* was written by the ethnographer and poet Victor Segalen (1878–1919), who integrated the psychological definition of synesthesia with certain stylistic devices of French symbolistic poets from the second half of the nineteenth century. (Millet had already mentioned them in 1892; a precursor to this was Petrich 1878, 13–39, who described the use of metaphors [i.e., “the color sounds”] in German poets of the early romanticism.) These poets drew connections between the senses, under the same term *synesthésie*: Charles Baudelaire (1821–1867), for example, had compared perfumes, colors, and musical tones in his poem “Correspondances” (1918); Arthur Rimbaud (1854–1891) linked vowels with colors, in his sonnet “Voyelles” (1972); Joris-Karl Huysmans (1848–1907) had associated liqueurs with musical instruments in *À Rebours* (Huysmans 1903, chap. 4, 48–50); René Ghil (1862–1925) had linked musical instruments with colors in his *Traité du verbe* (1886, 27). These were unlikely to have been synesthetes with involuntary additional sensations, but were instead looking for new means of expression within their poetry. Segalen was aware of the discussion from the nineteenth century in defining synesthesia and finding terms for the condition, but he integrated involuntary as well as voluntary combinations of senses, and initiated a discussion on the “*valeur artistique des synesthésies*” (artistic value of synesthesiae; Segalen 1902, 64).

From this point on, the term synesthesia was used not only for involuntary additional sensations, as we have defined this phenomenon at the beginning of this article, but also for the describing stylistic devices of poets, even outside of France, and later for correspondences within the arts in general. Concerning poets, some early examples of publications using the term in their titles serve to illustrate this development (Downey 1912; Fischer 1909; Fleischer 1911; Laures 1908; Margis 1910; Stock 1914; von Siebold 1919). The tendency to have separate definitions and uses of synesthesia in different disciplines—especially concerning literature, art, and music, which differ from the narrowed psychological definition—is an ongoing process still seen today (e.g., publications in the twenty-first century about correspondences of the arts with synesthesia in the title; Brougher et al. 2005; Udall and Weekly 2010; Wohler 2010; Che et al. 2012; Evers 2012; van Campen 2013; Cavallaro 2013; cf. Jewanski and Sidler 2006; Jewanski and Düchting 2009, 89–95; Jewanski 2018) and is outside the scope of the current article. We therefore provide two appendices that summarize the uses of the different terms and definitions we have carefully reviewed in our article.

## Notes

1. Note that the English translations of foreign language titles in text and references are literal and do not include different meanings of synesthesia, *audition colorée*, and similar terms, as explained in the main text (cf., for example, Binet 1893).

2. In an earlier translation (Jewanski, Day, and Ward 2009, 297; Jewanski, Ward, and Day 2012, 58; Jewanski, Ward, and Day 2014, 74), we used “dark ideas,” but “obscure ideas” seems to be more appropriate and fits better with Forster’s translation of obscure feeling.

## Appendix 1.

**Table:** Overview on the development of the various terms for the condition we today name synesthesia (1772–1892).

Year	Language	Original term	English translation of the original term	Source
1772	German	schnelle Anwandelung dunkles Gefühl	Fast onset obscure feeling	Herder (1772, 94–95) Herder (2002, 106)
1812	Latin	Latin (1812)/German (1824) phenomena/Erscheinungen obscura repraesentatio/dunkle Vorstellungen ipsam repraesentationem coloratam videri/daß ihm eine gefärbte Vorstellung erscheine	Features obscure ideas that a colored idea appears to him	Sachs (1812, 80–81) Sachs (1824, 99)
1813	German	farbige Erscheinung	Colored manifestation	Anonymous (1813, 236)
1814	German	gewisse Dinge als farbige Gegenstände auf eigene Weise repräsentirt	Represents special things as colored objects in his own way	Anonymous (1814, 12)
1848	French	hyperchromatopsie (perception de trop de couleurs)	Hyperchromatopsy (perception of too many colors)	Cornaz (1848, 150)
1864	French	pseudochromesthésie	Pseudochromesthesia	Chabalier (1864)
1873	English	idiosyncrasy		Hudson (1873)
1873	German	subjective *Farben*empfindungen subjective Farben-empfindungen	subjective *color* sensations	Nussbaumer (1873a) Nussbaumer (1873b)
1873	German	Phonopsie	Phonopsia	Nussbaumer (1873b, 60)
1879	English	Double sensation		Lewes (1879, 280–287)
1880	English	[seers]		Galton (1880b, 495)
1881	German	Secundärempfindungen Secundärvorstellungen	Secondary sensations secondary imaginations	Bleuler and Lehmann (1881, 3), note
1881	German	1881: Farbenhören 1881: English (US): color hearing 1881: English (UK): color-hearing 1882: French: audition colorée 1883: Italian: udizione colorata 1888: English/French: color-audition 1888: English: colored hearing 1890: Swedish: färghörsel 1893: English/French: colored audition 1894: Russian: воспринимание слухом цветов 1902: Spanish: audición coloreada	Color(ed) hearing	Anonymous (1881a) Anonymous (1881c) Anonymous (1881d) Pédrono (1882a, 1882b) Grazzi (1883) Color-audition (1888) Billings (1888, 60) Wahlstedt (1890) Binet (1893) Nordau (1894, 144) Nordau (1902, 218)
1884	German	Mitempfindung	Co-sensation	Hilbert (1884, 3)
1887	German	secundäre Sinnesempfindungen	Secondary sensations of senses	Steinbrügge (1887)
1889	German	secundäre Empfindungen	Secondary sensations	Deichmann (1889)
1889	German	Doppelempfindung (Secundärempfindung)	Double sensations (secondary sensation)	Ellinger (1889)
1889	French	chromatopsie	Chromatopsia	Sperling, cited in Société de psychologie physiologique (1890, 95)
1890	French	fausse sensations secondaire	False secondary sensations	Suarez de Mendoza (1890, 8)
1892	French English	synesthésie synæthesiæ	Syn(a)esthesia	Millet (1892, 13) Myers (1892, 457)

The sources are “latest dates” and perhaps can be antedated by further research.

## Appendix 2.

**Table:** First appearances of the term “synesthésie” (or variants) in our modern meaning in different languages and different spellings.

Year	Language	Term	in an abstract/a review	(Hidden) inside an article or a monograph with a different title	as a title of an article
1892	French	synesthésie		Millet (1892, 13) Flournoy (1893, 6)	Segalen (1902)
1892	English	synæsthesia, synesthesia, synesthesia	Calkins, 1893b (review on Flournoy 1893)	Myers (1892, 457) Colman (1894, 795 and 851)	Calkins (1895)
1894	Italian	sinestesia		Pilo (1894, 140)	Pilo (1894) (fenomeni sinestesici) Mirto (1894)
1894	German	Synaesthesie, Synästhesie	Barth (1895) (review on Colman 1894) Cohn (1895) (review on Flournoy 1893) Hager (1895) (review on Mirto 1894) Ziehen (1895) (review on Colman 1894)	Parish (1894, 156)	Stelzner (1903)
1897	Russian	Синестезия		Sokolov (1897, 253)	Ermakov (1914)
1908	Spanish	sinestesia		Mercante (1908, 422)	Beltrán (1931)

The sources are “latest dates” and perhaps can be antedated by further research.
